# Natural disasters and SARS-CoV-2: potential risk factors for exacerbating mental health conditions among veterans

**DOI:** 10.1007/s44192-025-00147-z

**Published:** 2025-03-04

**Authors:** Parthasarathy Arpitha, Laura Bolorin-Vargas, Glorisel Gonzalez-Viera, Marta Rodriguez-Garcia, Gerardo Jovet-Toledo, Irma L. Molina-Vicenty, Luis Collazo-Rodriguez, Maria Leticia Reyes-Rabanillo

**Affiliations:** 1 Veterans Affairs Caribbean Healthcare System, 10 Calle Casia, San Juan, Puerto Rico 00921 USA; 2 Ponce Health Science University, 388 Zona Industrial Reparada 2, Ponce, Puerto Rico 00716 USA

**Keywords:** Mental health exacerbation, COVID-19, SUD, PTSD, Natural disasters, Mental health

## Abstract

**Background:**

Puerto Rico and U.S. Virgin Islands Veterans (PRVs) have faced recurring challenges from hurricanes, earthquakes, and the COVID-19 pandemic. These events combined with prior traumas and social determinants of health (SDoH), may contribute to neuropsychiatric mental health conditions (MHCs) like post-traumatic stress disorder (PTSD) and substance use disorder (SUD) in PRVs affected by SARS-CoV-2.

**Methods:**

To clinically characterize the risk factors for MHCs among SARS-CoV-2 infected PRVs, we examined 839 records (2016–2020) to study SARS-CoV-2 infections and MHCs. Records were assessed for (i) PTSD and SUD using health data at diagnosis; (ii) clinical details pre-hurricanes (control; group 1), during hurricanes (group 2), and pandemic (group 3). Groups 1 and 2 were reviewed in 2020 to gauge exacerbation. Patient Health Questionnaire (PHQ-2/PHQ-9), PTSD checklist (PCL-5), Alcohol Use Disorders Identification Test (AUDIT), SDoH, and other tools were used for clinical evaluation, with the data analyzed using logistic regression.

**Results:**

Health data indicated SARS-CoV-2 infection in 21 PRVs. Earthquakes did not affect the infected PRVs nor did SDoH have any significant impact. Clinical analysis revealed that SUD worsened during hurricanes and exacerbation of all MHCs occurred during the pandemic among SARS-CoV-2 infected PRVs.

**Conclusions:**

These results underscore the fact that the combination of natural disasters like hurricanes and SARS-CoV-2 had synergistically contributed to the deterioration of neuropsychiatric MHCs, therefore warranting equitable MH support.

## Introduction

The global COVID-19 pandemic (hereafter referred to as ‘pandemic’), caused by the respiratory virus SARS-CoV-2, has also had an impact on the neuropsychological and neuropsychiatric clinical symptoms within the U.S. population, giving rise to increased public health concerns. In the U.S., the pandemic has resulted in over a million deaths and a surge in mental health conditions (MHCs) [1, 2]. Some patients infected with SARS-CoV-2 have been reported to experience dementia, altered mental status, and new early-onset psychosis, as documented by UK researchers at the CoroNerve Studies Group [3]. Recent findings indicate that the SARS-CoV-2 virus leads to brain inflammation [4], encompassing conditions such as encephalopathy or encephalitis [3]. Alongside the impact of the pandemic on MHCs, the prevalence of mass fatalities and health disparities among marginalized populations has brought about changes in the global economy.

Currently, there is insufficient health economic data available to explain whether social determinants of health (SDoH) can influence an individual’s MHCs and/or propensity towards suicidality due to the economic disparities resulting from the pandemic. Notably, Puerto Rico has emerged as one of the top five locations in the U.S. with high rates of suicidality during the pandemic [5].

Veterans represent a unique sector of our society that requires more healthcare attention due to their profession and past combat experiences, which make them highly predisposed to mental health and emotional challenges. Additionally, some struggle to adapt to new changes when transitioning to civilian life. Veterans form a highly vulnerable population that is susceptible to conditions such as post-traumatic stress disorder (PTSD) and concurrent substance use disorder (SUD), with a reciprocal relationship [6–9] including the risk of suicidality. Since 2020, the ongoing pandemic has impacted Veterans who are predisposed to neuropsychiatric symptoms, including PTSD [10].

The psychological and immunological impact of PTSD caused by the pandemic has been well-documented. This impact has been reported worldwide, including in countries such as Italy [11], China [12], France [13], the UK [14], and the U.S. [15]. The outcome of these studies has suggested that the repercussions of the pandemic have led to a neuropsychiatric sequence of events, resulting in an increase in PTSD followed by immune suppression, thus deteriorating the quality of life [1, 7–9, 15]. Many of these patients present with increased distress and a higher rate of hospitalization for various medical and psychiatric symptoms, including PTSD [16] and display suicide risk factors that could lead to increased rates of suicide [17].

The rate of attrition, job loss, and social distancing, among other factors associated with the pandemic, may lead to an unprecedented increase in SUD and PTSD among Veterans who may be impacted by the social determinants of health [18, 19]. One way to identify the risk factors for MHCs is based on five parameters of SDoH [20] to investigate the aftereffects of a neuropsychiatric diagnosis.

The Veterans Affairs Caribbean Healthcare System (VACHS), which provides services to Puerto Rico and the U.S. Virgin Islands, caters to patients who are more susceptible to health disparities, have faced environmental-associated disasters, such as Hurricanes Irma and Maria in 2017 [21] earthquake in January 2020, and the pandemic in March 2020. Exposure to these natural disasters has been considered life-threatening events that can lead to Post-Traumatic Stress Disorder (PTSD) and other MHCs [22]. In recent years, an increase in Puerto Rican Veterans (PRVs) suicide rates was observed (Reyes-Rabanillo, 2020, unpublished data). Most individuals who commit suicide may have MHCs, including PTSD, Substance Use Disorder (SUD), or a dual diagnosis with both comorbidities. PTSD may be exacerbated by withdrawal and other risky behavioral patterns in SUD patients [23]. Additionally, PTSD has been suggested to precede substance use to alleviate the symptoms of stress [24], giving rise to the dual diagnosis. The concept of dual diagnosis is considerably intricate and therefore could potentially be one of the risk factors contributing to the increased prevalence of suicide, a less explored area in precision psychiatry.

Considering the reported increase in the number of suicides among Veterans in recent months [17] including VACHS Veterans (Reyes-Rabanillo, 2020, unpublished data), we hypothesize that SARS-CoV-2 infection among PRVs who were impacted by hurricanes and the mental health challenges associated with the pandemic may exacerbate clinical neuropsychiatric symptoms related to PTSD and SUD. As far as our knowledge extends, the present study is the first to identify and clinically characterize the risk factors for PTSD and SUD in association with SDoH among SARS-CoV-2 infected PRVs.

## Methods

### Study population

The project at VACHS received institutional review board (IRB) approval to conduct this study and retrieve data from Health Information Management Services (HIMS) for the study population. Since this study involved a retrospective record review and did not require the recruitment of Veteran participants, no consent for data usage from participants was sought, nor was ethics approval considered necessary. This study obtained approval from the institutional review board of VACHS (Project No. 1599779). The study focused on PRVs which also included US Virgin Island subjects (none diagnosed with SARS-CoV-2) with MHCs like PTSD and SUD including those who committed suicide between 2016 and 2020.

**Fig. 1:**
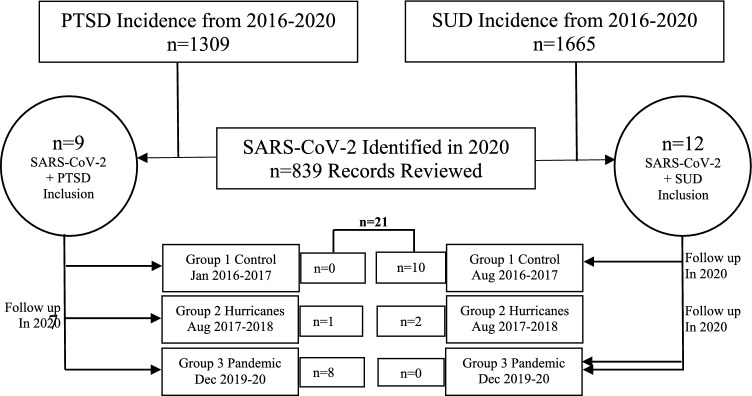
Study population presenting with PTSD and SUD with SARS-CoV-2 at VACHS. Table 1 shows the incidence and population of PTSD and SUD during 2016–2020 and how we arrived at the inclusion of the study population of n = 21 who presented PRVs with a diagnosis of PTSD or SUD along with SARS-CoV-2 infection. Post-traumatic stress disorder (PTSD), substance use disorder (SUD), Veterans Affairs Caribbean Healthcare System (VACHS), Puerto Rican Veterans (PRVs)

The epidemiological incidence of newly reported MHCs between 2016 and 2020 was examined. We found n = 1309 cases diagnosed with PTSD and n = 1665 cases diagnosed with SUD. The patient identifiers were obtained from HIMS-Veterans Affairs records to verify their diagnoses. To identify the specific subjects included in this study, records were reviewed to identify PRVs with SARS-CoV-2 infection (n = 839 identified in 2020). These subjects were then cross-referenced for their PTSD and SUD diagnoses, ensuring that they were also among the n = 1309 PTSD and n = 1665 SUD cases confirmed by HIMS records. This process resulted in the identification of n = 21 inclusive cases (Fig. 1). Health informatics and clinical characterization of these cases were performed within three distinct time frames:

1. The period between January 2016 and January 2017, represented the non-calamity control period (group 1) before the occurrence of the hurricanes.
2. The period between August 2017 and August 2018, covered the two Puerto Rican hurricanes, Irma and Maria (group 2).
3. The period between December 2019 and December 2020, during the pandemic (group 3) (Fig. 1).

To summarize, Group 3 consisted of newly diagnosed subjects with MHCs in 2020 who were also infected with SARS-CoV-2. On the other hand, Group 1 and Group 2 included subjects who had a history of MHCs and were also infected with SARS-CoV-2 in 2020. A retrospective review of the Computerized Patient Record System (CPRS) medical records was conducted for subjects in groups 1–3, establishing a "baseline expression." Only subjects from groups 1 and 2 were reviewed again during the pandemic (from January 2020 to December 2020) and were referred to as "follow-up" cases. The data collected from these reviews were categorized and analyzed in the present study under two main sections: health informatics and clinical characterization.

### Inclusion and exclusion criteria

Only PRVs who tested positive for SARS-CoV-2 and presented for the first time with MHCs, without a past medical record before the study period (groups 1–3), were included in this study. Both male and female Veterans above the age of 21 years were included, while SARS-CoV-2 negative cases were excluded.

### Health informatics

The analysis of health informatics focused on ten parameters that encompassed past combat status, addiction history, medical conditions presented when the PRVs were first diagnosed with MHCs, history of other psychiatric illnesses, and demographic status that may have influenced their geospatial health and epidemiological incidence of PTSD and SUD. Additionally, demographics data and deployment status were collected, along with health conditions and past and present medical history when the PRVs were first diagnosed with PTSD and SUD.

### Clinical characterization of mental health conditions among SARS-CoV-2 infected subjects

Subjects were retrospectively reviewed for twenty-four parameters based on clinical documentation recorded by suicide prevention case managers, psychiatrists, primary care physicians, and psychologists. Risk for suicide was clinically assessed using PTSD scales from DSM-5 (CAPS-5) and PTSD Checklist (PCL-5), as well as the Brief Addiction Monitor (BAM) and AUDIT measurements (at the SUD clinic). If clinical scales were not available, we incorporated the physician's decision as confirmation for diagnosis.

Other parameters included the number of hospital readmissions, telehealth consults, treatments for suicidality, deaths, consults for PTSD and SUD, including suicidal ideations and suicidal attempts, trust difficulties, insomnia, global guilt score, self-rated health problems, SDoH, and the 7-factor PTSD Hybrid Model (20 parameters as described by [25–27]. SDoH was categorically extrapolated for SARS-COV-2 positive (groups 1–3) from CPRS for the five parameters: (a) healthcare access, (b) education, (c) social community context, (d) economic stability, and (e) neighborhood/nurtured environment, including access to food, water, and the influence of the crime rate in the neighborhood [18–20]. For every variable, a score of + 1, 0, or − 1 was ascribed and recorded for further data analysis. A higher average score for SDoH parameters indicated the presence of protective factors, and a lower score indicated a worsening of SDoH conditions.

Data were analyzed by comparing the inter-groups (e.g., SUD in group 1 compared to SUD in group 2) and intra-groups for the same MHCs (e.g., the same patient in SUD in group 1 compared to SUD in 2020; a patient presenting an MHCs for the first time was analyzed as a baseline versus follow-up of the same patient in 2020). A follow-up record review for the same PRVs was carried out in 2020 for SARS-CoV-2 infected subjects in groups 1 and 2 after four and three years, respectively to clarify whether there was an improvement in health outcomes after treatments or not.

### Exacerbation of clinical symptoms

Groups 1 and 2 were compared with group 3, meaning that control samples and hurricane-affected individuals were compared with those affected during the pandemic period to estimate exacerbation. Subjects were assessed for all 24 clinical parameters in 2020, and the records were examined to determine if they were influenced by the earthquakes.

### Statistics

To prevent any discrepancies among the data collectors from CPRS, Cohen’s Kappa was conducted using SPSS software (IBM Armonk, NY, USA). Data analysis was based on composite analysis using 24 parameters for clinical characterization and 10 parameters for health informatics for the two MHCs within the three groups. Statistical analysis was performed using logistic regression at a 95% confidence interval (Microsoft, Seattle, USA). If the regression R2 did not fit the model due to low sample availability constraints, as per the nature of this study, and/or due to the availability of SARS-CoV-2 positive cases with MHCs, a student t-test was used to interpret the data at P ≤ 0.05.

## Results

### Suicidality and underlying mental health diagnosis in the study population

At VACHS, there was a large population of Veterans presenting with PTSD and SUD. However, upon reviewing the records for actual cases that had both viral infection and MHCs, we found only n = 21 cases that presented with SARS-CoV-2 along with PTSD or SUD (Fig. 1). While examining the records for various variables and data collected by multiple collectors in the study for the n = 21 cases (a total of 9 cases with PTSD were identified in groups 2 and 3, and a total of 12 SUD cases were identified in groups 1 and 2), we confirmed that Cohen’s kappa values agreed. Cohen’s kappa scores for the data collectors ranged from 0.41 to 0.60, indicating moderate agreement; 0.61 to 0.80, indicating substantial agreement; and 0.81 to 1.00, indicating almost nearly perfect agreement. This allowed us to proceed with further data collection.

### Incidence and health assessment of veterans presenting with an MHC for the first time

The results presented in Fig. 1 prompted us to conduct a comprehensive study on the two MHCs, namely PTSD and SUD, between 2016 and 2020. A total of eight hundred and thirty-nine male and female individuals aged 43 to 58 years, referred to as PRVs, presented with SARS-CoV-2 infection between December 2019 and November 2020 (Table 1). Among these PRVs, only 4.05% had been diagnosed with MHCs before natural calamities, such as hurricanes, and during the pandemic. This finding confirms the epidemiological incidence at VACHS. In other words, the sub-population of PRVs that had MHCs such as PTSD or SUD in conjunction with SARS-CoV-2 infection accounted for a total of n = 21 individuals (Fig. 1). All PRVs included in this study had a regular discharge from their service. Within this population, only 1% of individuals had other psychiatric conditions (e.g., dementia; referred to as group 1), and none were affected by the earthquakes.

**Table 1:** Health informatics of subjects infected with SARS-CoV-2 with underlying mental health conditions during Puerto Rican catastrophes

Category	Sub-category (data presented as mean % for each parameter)	Group 1 SUD (n = 10)	Group 2 PTSD (n = 1)	Group 2 SUD (n = 2)	Group 3 PTSD (n = 8)
Gender	Sex (M/F)	M	M	1 M, 1 F	2F, 6 M
Age	Age	58.8	43	43	47
Combat status	Combat/work-related	53.06	70	0	61.47
	Non-combat or not work-related	46.93	0	87.5	38.2
	Gulf war combat-related	53.06	70	0	61.47
Addiction Hx	Tobacco use	42.8	100	50	12.5
	Other substance use	20	0	0	0
	Alcohol addiction	70	0	0	0
Med Hx	Diabetic	30	0	0	0
	HTN	70	0	0	37.5
	CAD	20	0	0	12.5
	Lung disease	0	0	0	12.5
	Hypercholesterolemia/hyperlipidemia	40	100	50	37.5
	Other past Med Hx	100	0	100	62.5
	Neurological disease (other than depression/PTSD)	100	100	50	100
	History of cancer	30	0	0	20
	Other co-morbidities	70	100	0	0
	Surgical history	10	100	50	0
	No. of hospitalization/subject for other co-morbidities	12	100	0	25
History of other current/past psychiatric illnesses	Psychiatric depression	50	0	50	62.5
	Other MH conditions (dementia, Alzheimer’s, etc.)	30	0	0	0
	PTSD	20	0	0	12.5
	None	20	100	0	0
	Current psychosis or mania	10	0	0	37.5
	Undergoing psychological counseling for trauma	20	0	50	87.5
	Currently on trauma-focused treatment like PE or CPT	0	100	0	25
	Sleep disturbances/sleep apnea	57.1	0	50	75
	Life-threatening unstable medical illness	40	0	0	0
	Killing enemy combatants	0	100	0	0
	Unjust war events	10	100	0	0
	Life-threatening events	20	0	0	75
	War injury and traumatic experience & guilt	0	0	0	12.5
	Traumatic brain injury screened positive for TBI	0	0	0	0
Geographic distribution (%)	Rural demographics	0	0	0	0
	Urban demographics	100	100	100	100
	Residence > 50miles from the nearest study site	28.5	0	0	0
Job status (%)	Employed	30	0	0	25
	Unemployed	40	0	100	12.5
	Retired	10	0	0	62.5
	Unknown	20	100	50	0
Education	High school	30	100	0	12.5
	Under graduation	40	0	0	50
	Post-graduation	10	0	50	12.5
	Doctorate	0	0	0	12.5
	Unknown	20	0	50	0
Ethnicity/	Hispanic/Latino	100	100	100	75
Race	African American	10	0	0	25
	Caucasian	90	100	100	75
Health informatics	(% mean of the mean)	* 34.1 ± 27.9	** 35.2 ± 46.8	* 22.9 ± 29.2	** 28.4 ± 0.3

Ten parameters were analyzed for groups 1–3 to assess health informatics status. Data was presented as % average, while statistical analyses were based on goodness of fit for regression among the MHCs

Post-traumatic stress disorder (PTSD), hypertension (HTN), cardiac disease (CAD), prolonged exposure (PE), cognitive processing therapy (CPT), and traumatic brain injury (TBI). R2 = 58.6%, *P < 0.001 for SUD compared between groups 1 and 2; R2 = 50.01% and **P < 0.001 for PTSD compared between groups 2 and 3. Note, statistical significance identified as ^*, **^ will appear twice in the table to indicate the two parameters that were used for statistical comparison; data are presented as average for each parameter, statistical regression R^2^ was considered fit if P ≤ 0.05

The overall PRVs health assessment for each of the MHCs were scored based on composite factors, which included combat status, addiction history, medical history, psychiatric history, and geographic status (including the level of education, employment history, race, and ethnicity—as shown in Table 1). The epidemiological incidence of PRVs with SUD in group 1 (Jan 2016–2017), who had SARS-CoV-2 infection in 2020, was observed only during the non-calamity period. However, during the hurricanes, MH diagnoses for PTSD and SUD were predominant in group 2 (Aug 2017–2018) among those with SARS-COV-2 infection in 2020 (Table 1).

In the study period before the hurricanes, 29.4% of PRVs presented with SUD, and during the hurricanes, 2.94% of them presented with PTSD, and 5.88% presented with SUD. On the other hand, newly diagnosed PTSD cases were found during the pandemic in group 3 (Dec 2019 to 2020) among PRVs with confirmed SARS-CoV-2 infection. Notably, the incidence of MHCs was not the same across the three study periods (Table 1).

To further comprehend the variation in the incidence of MHCs and geospatial health across the three PRVs groups, the scores (%) for all ten informatics parameters (excluding age and gender identity) were averaged and subjected to statistical analysis. As depicted in Table 1, a noteworthy increase (P ≤ 0.05) in the health informatics score was evident among individuals in group 1 (34.05 ± 27.92) in comparison to group 2 (22.90 ± 29.22) for SUD. Similarly, subjects in group 2 (35.17 ± 46.80) displayed a higher score than group 3 (28.40 ± 30.26) for PTSD. In principle, a significant disparity in the overall geospatial population health status was discernible (considering all presenting MHCs during the control period, hurricanes, and pandemic). This disparity manifested as improved health for SUD subjects during the pandemic, whereas deteriorating health conditions were noted for PTSD during the hurricanes when initially observed at VACHS.

### Clinical characterization and exacerbation of clinical symptoms during hurricanes and the pandemic

A composite analysis was employed to characterize the clinical aspects of SARS-CoV-2 infected PRVs concerning their MHCs (PTSD, SUD) using twenty-four parameters, including SDoH and a 7-factor hybrid PTSD model [27]. No statistical significance was identified among SUD in groups 1 and 2 or among PTSD in groups 2 and 3. However, most reported traumas were linked to war injuries, deaths, witnessing horrifying events, or experiencing fear during military service (53.6% in group 1; 70% in group 2; and 64.43% in group 3, Table 1). Additionally, nearly all PRVs exhibited mild to moderate PTSD and varying degrees of depression (Table 2). PTSD symptoms significantly escalated during the pandemic compared to the hurricane period for the same patients. Moreover, PRVs newly diagnosed with PTSD who were infected with SARS-CoV-2 (group 2 baseline and/or group 2 follow-up versus group 3; P ≤ 0.05, P ≤ 0.001, respectively, Table 2) displayed noteworthy exacerbation compared to subjects affected only by the hurricane (group 2 baseline versus follow-up, P ≤ 0.05, Table 2). Both PTSD and SUD significantly increased during the hurricanes and the pandemic (P ≤ 0.05, Table 2). The same subjects in groups 1 and 2 exhibited worsening mental health clinical symptoms two years later at the time of the 2020 follow-up.

**Table 2:** Clinical characterization of mental health conditions among SARS-CoV-2 infected subjects showing exacerbation in their symptoms

Group 1 (non-calamity baseline period), Group 2 (Hurricanes) and Group 3 (Pandemic)	Group 1 SUD	Group 1 SUD	Group 2 PTSD	Group 2 PTSD	Group 2 SUD	Group 2 SUD	Group 3 PTSD
Baseline and follow-up of the same case during the pandemic; statistical regression compared within each disease condition among Group 1–3	Non-calamity/baseline ^*, a, b^	3 yr Follow-up of Group1 SUD during pandemic ^*, c, d^	During hurricanes/baseline ***	2 yr Follow-up of Group 2 PTSD during pandemic ***, ^e^	During hurricanes/baseline ^∫, a, c^	2 yr Follow-up of Group 2 SUD during pandemic ^∫, b, d^	Baseline during pandemic ^†, e^ (data post pandemic not available)
No. of subjects in each group (Total SARS-CoV-2 positive cases = 21)	10	10	1	1	2	2	8
Data is presented as the average number per subject for each parameter Statistical regression and P-value were compared within Groups 1 to 3	Group 1 SUD baseline vs follow-up R^2^ = 90.77%; P < 0.001*		Group 2 PTSD baseline vs follow-up T-Test P < 0.005***		Group 1 vs 2 SUD R^2^ = 34.6%; P < 0.0001^∫^ R^2^ = 22.9%; P < 0.001a R^2^ = 28.5%; P < 0.0001b R^2^ = 28.58%; P < 0.0001c R^2^ = 20.03%; P < 0.005d		Group 2 vs 3 baseline PTSD R^2^ = 8.75%; P < 0.05†R^2^ = 36.04% P < 0.0001e
Hospital readmissions	0.1	0.125	0	0	0	0.67	0.25
Telehealth consults	1.3	0.75	1	5	1	9.67	2.88
Treatments for suicidality	0.1	0.125	4	0	0	0	0.5
Deaths—natural or suicide	0	0	0	0	0	0	0
Consult for PTSD	0	0	4	1	0.5	0	2
Consult for SUD	0.4	0.5	2	0	0	0	1
Consult for PTSD-SUD	0.3	0.375	2	0	0.5	0	0
Consult for high-risk suicidal ideations	0.1	0.125	2	0	0	0	0.13
Consult for high-risk suicidal attempts	0.1	0.125	0	0	0	0	0
DSM-5 (CAPS-5) score	0	0	1	0	0	0	15.33
PHQ-2 depression score	0.2	0.125	1	0	3	0.67	0.25
PHQ-9 depression score	0.9	1.125	1	18	13	6	11.63
BAM score	1	1.25	1	0	0	0	0
AUDIT score	0.9	1.125	4	0	1.5	1.33	0.38
Columbia-suicide severity score	0	0	0	0	0	0	0
Generalized anxiety disorder score	0	0	0	0	0	5.67	6.4
Trust difficulties	0.2	0	1	0.5	0	0	0
Insomnia	0.4	0.375	0	1	0.5	0.67	0.63
Global guilt score	0	0	1	0	0	0	0
Self-rated behavioral health problems	0.9	1	0	1	0	0.67	1
Seclusion due to the Pandemic and lack of healthcare access	0	0	1	0	0	0	0
Social determinants of health	3.67	3.86	2	5	5	5	1.875
Average score for 7-factor PTSD hybrid model (20 parameters)	0.6375	0.933	0.5	0.9	0.35	0.2	0.8055

Clinical symptoms of PTSD, SUD wand ere significantly exacerbated for the same Veteran during the Pandemic. Note, that statistical significance identified as ^*, **, ***, a, b, c, d, e, ∫, †^ will appear twice in the table to indicate the two parameters that were used for statistical comparison; data are presented as average for each parameter, statistical regression R^2^ was considered fit if P ≤ 0.05. *Abbreviations:* Post-traumatic stress disorder (PTSD), substance use disorder (SUD), patient health questionnaire (PHQ-2/PHQ-9); PTSD checklist (PCL-5), diagnostic and statistical manual 5 (DSM5), alcohol use disorders identification test (AUDIT), brief addiction monitor (BAM)

We applied the 7-factor hybrid PTSD model proposed by Claycomb et al. [27] to both PTSD and SUD groups (n = 21). This model is a modified version of the Anhedonia model [25] and the externalizing behavior model [26]. The clinical characteristics of individuals with PTSD, encompassing seven PTSD factors (intrusions, avoidance, negative affect, anhedonia, externalizing behaviors, anxious arousal, and dysphoric arousal), were found to be embedded among those with SUD, as evidenced in Tables 2 and 3. When we applied the 7-factor hybrid PTSD model to the data collected from CPRS, we observed that PRVs diagnosed with SUD exhibited clinical symptoms of PTSD. The mean 7-factor hybrid score for PTSD using the 7-factor hybrid analysis was 56%, while the embedded PTSD score for SUD was 4.36%. The baseline expression of this embedded PTSD diagnosis within SUD was significantly exacerbated during the hurricanes and the pandemic (Table 3).

**Table 3:** 7-Factor hybrid PTSD model showing exacerbation symptoms

7-Factor hybrid model of PTSD (% Average No. of subjects)	Group 2 PTSD Baseline *	Group 2 PTSD Follow-up *, ^†^	Group 3 PTSD Baseline *, ^†^	Group 1 SUD Baseline **, ^††^	Group 1 SUD Follow-up **, ^∫^, ^∮^	Group 2 SUD Baseline ^††, ∫^	Group 2 SUD Follow-up ^††, ∮^
Statistical Regression R^2^/T-test and P < 0.05 compared within each disease condition among Group 1–3	T-Test P < 0.05 *	T-Test P < 0.05 *	R^2^ = 76.8% P < 0.001 ^†^	R^2^ = 25.2% P < 0.005 ^††^	R^2^ = 84.5% P < 0.001 **	R^2^ = 34.3%P < 0.005 ^∫^	R^2^ = 18.1% P < 0.005 ^∮^
1. Intrusion factor (intrusive thoughts, nightmares, flashbacks, emotional & physiological cue reactivity	40	100	9.725	7.5	10	0	0
2. Avoidance factor (avoidance of thoughts & reminders)	100	50	7.875	5	10	25	0
3. Negative Affect (trauma-related amnesia, negative beliefs, blame, negative trauma	25	100	11.0625	1	10	12.5	0
4. Anhedonia factor (loss of interest, detachment, restricted effect)	33	100	11.5	5.8	10	50	16.5
5. Externalizing Behavioral factors (irritability, anger, self-destructive)	50	50	6.6875	1.25	0.5	0	25
6. Anxious Arousal factor (hypervigilance, exaggerated startled response)	100	100	12.5	5	10	0	0
7. Dysphoric Arousal factor (difficulty concentrating, sleep disturbances)	50	100	10	5	8.3	50	50
% Average score for 7-factor Hybrid model of PTSD (20 parameters)	56	85	9.91	4.36	8.4	19.64	13.07

This table demonstrates the use of 7 factors being applied for analyzing mental health conditions to identify PTSD characteristics and its exacerbation. Data presented as % average for all the seven factors in each group; statistical regression R^2^ was considered fit if P ≤ 0.05. Note that statistical significance represented as ^*, **, ∫, †, ǂ, ∮^ will appear twice in the table to indicate the two parameters that were used for statistical comparison

Post-traumatic stress disorder (PTSD), substance use disorder (SUD)

To gain a deeper understanding of the underlying risk factors that may have contributed to mental health exacerbation, we conducted independent analyses of the five factors of SDoH for all 21 SARS-CoV-2 infected individuals within the three groups. As shown in Table 4, there was a significant improvement in SDoH among those with substance use disorder (SUD) (P ≤ 0.001) when compared to the control period. There were no notable differences in SDoH for subjects with Post-Traumatic Stress Disorder (PTSD); however, their clinical symptoms worsened (Table 2). The exacerbation of PTSD may be attributed to underlying risk factors such as their past combat/military service experience (Table 4) and the compounded impact of hurricanes and the COVID-19 pandemic. Additionally, we found that past military service or combat experience was not associated with an SUD diagnosis (P ≤ 0.05), and the PRVs in the SUD group exhibited improvements in SDoH during the pandemic (Table 4). Throughout this period, there were two deaths; however, none were attributed to suicide but rather to other comorbidities. The PRVs who passed away due to pneumonia and other health complications had a diagnosis of SUD at the time of their demise (Table 4).

**Table 4:**
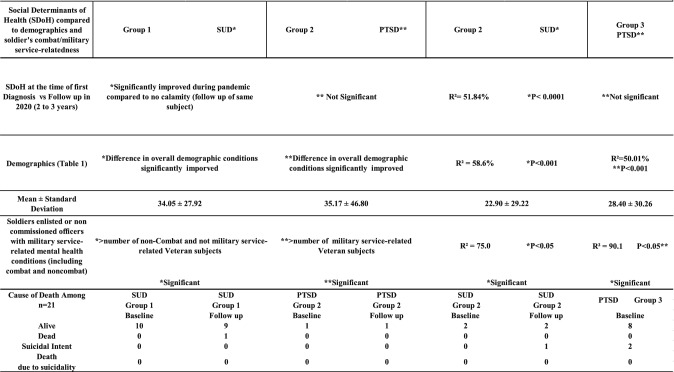
Characterization of social determinants of health and suicidality among SARS-CoV-2 positive mental health subjects

The analysis for SDoH was based on military service or combat-relatedness and health informatics during Puerto Rican hurricanes and the COVID-19 Pandemic. Note that statistical significance identified as ^*, **^ appears twice in the table to indicate the two parameters used for statistical comparison

Post-traumatic stress disorder (PTSD), substance use disorder (SUD), and Social Determinants of Health (SDoH). The table also shows the mental health conditions related to suicidal ideations

## Discussion

Herein, we report that the Puerto Rican natural catastrophes have emerged as potential environmental threats, increasing the exacerbation of mental health issues and posing a public health hazard for our Veterans. This study presents, for the first time, findings that PRVs infected with the SARS-CoV-2 respiratory virus and diagnosed with existing comorbid MHCs during the Puerto Rican hurricanes in 2017, the early 2020 earthquakes (none of these cases had concurrent SARS-CoV-2 infection along with PTSD or SUD), and the ongoing pandemic, have exhibited exacerbated mental health symptoms associated with PTSD and SUD. These symptoms can lead to a high risk for suicidality (HRS).

Remarkably, the follow-up test, which aimed to assess the recovery of Veteran’s MHCs after 3 to 4 years of treatment, unexpectedly demonstrated a worsening of these conditions. This highlights the severe impact of natural disasters and their ramifications, including but not limited to the absence of mental health clinic access and care, challenges in medication compliance, and potential effects on Veteran retention, which may have contributed to an increased burden on mental health. Furthermore, given that a substantial proportion of suicide completions were linked to individuals diagnosed with major MHCs of PTSD (12.9%), SUD (12.9%), dual diagnosis, and depressive disorders (data not shown), it became imperative for us to investigate the two primary MHCs, namely PTSD and SUD, in conjunction with SARS-CoV-2 infection.

From an epidemiological perspective, there was a significant breakthrough in SARS-CoV-2 infection among PRVs with MHCs during the pandemic (4.12%), which heightened the risk of exacerbating MHCs during the hurricanes (26.5%) in comparison to the control group (5.91%). These findings suggest that the exacerbation of MHCs was influenced by both the hurricanes and the pandemic among PRVs. These results were consistent with civilian population health studies [28, 29], which support our findings that SARS-CoV-2 infected subjects are at a risk for psychiatric conditions, and similar effects have been reported from hurricanes such as Katrina in the US [30–32]. Additionally, a recent study has also confirmed that survivors of SARS-CoV-2 infections experienced neurocognitive mental health deficits [33] that corroborated with the current study. Furthermore, at VACHS, although a total of N = 7961 Veterans received at least one dose of the COVID-19 vaccine between 2020 and 2024, accounting for 87.42% of vaccinated PRV, the island of Puerto Rico has reported infection rates up to 41% in 2024 to date (infection control, Departamento de Salud, Puerto Rico). This underscores the need for prospective clinical studies to mitigate long and recurrent infections and their effects on MHCs. Recent studies have indicated that infection-associated deaths have increased among elderly Veterans [34]. Moreover, published literature corroborates our findings that the aftermath of the pandemic and infections trigger neuropsychiatric risks, including suicide [35–38].

Furthermore, the application of the 7-factor hybrid PTSD method [25–27] in the present study proved effective in clinically characterizing PTSD symptoms among other MHCs that might otherwise have gone unnoticed. In general, our results align with recent findings that the pandemic has exacerbated PTSD symptoms in the civilian population[35, 38, 39]. More specifically, however, we have demonstrated for the first time that PTSD was detected in individuals diagnosed with substance use disorder (SUD), with PTSD prevalence ranging from 4.36 to 19.64%.

Despite the exacerbation of all MHCs during the pandemic, our findings indicate that the 7-hybrid PTSD characteristics accounted for only 9.91% of cases. At the same time, SUD exhibited recovery in SDoH during the pandemic.

This paradox could be elucidated by the socioeconomic support provided by the government at a national level during the pandemic, which may have acted as a protective factor through SDoH, reducing the risk of global and nationwide suicidality [5]. We are reporting, for the first time, that MHCs such as PTSD and SUD are associated with predisposing risk factors for suicidality among individuals infected with SARS-CoV-2 in the PRVs population.

Although the rate of suicidality had globally and nationally reduced, as reported by Prof Jane Pirkis in 2021, Puerto Rico remained among the top five regions in the U.S. with higher rates of suicide. Notably, the number of PRVs who committed suicide has progressively increased in the past four years following the natural catastrophes in Puerto Rico (data not shown). Suicide poses a philosophical and ethical dilemma since some may not agree that it is a mental health disorder, such as Sanati [40], due to the absence of precise measures such as biological markers which are not yet available. However, the present study and other clinical researchers support the idea that suicidality is still considered a mental health issue [41, 42]. This notion applies to Veterans given the nature of their grievances, socio-economic challenges, and SDoH, which have contributed to the progression of mental illnesses.

The lack of vaccination among individuals with mental health conditions could also pose a public health concern for suicide prevention [36], with the risk escalating among Veterans [27], including the vulnerable Veteran population. Several reports now emphasize the need to improve SDoH [35] and provide resources to address the long-term mental health effects of the pandemic on civilian populations [37]. These findings align with our clinical data showing that, despite the federal government providing iPad and phones for remote monitoring and telehealth to improve the mental health of PRVs, the suicide rate did not decrease in 2020 (data not shown). This correlates with unexpectedly high suicide rates in the Puerto Rican civilian population compared to other U.S. states [5]. Recent findings show that long-term exposure to SARS-CoV-2 is associated with higher rates of depression, sociopsychological disorders, and suicidal ideation, regardless of prior depressive symptoms [43]. Among aging PRVs, this exposure, combined with mental health comorbidities, may lead to unexpected mortality (Table 4). Therefore, follow-up care for all SARS-CoV-2-infected individuals at mental health/behavioral health clinics is crucial for treatment and suicide prevention.

SDoH has also been used as a mechanism to identify whether low socioeconomic status impacts MHCs for those at the lower end of the social gradient, including stress from routine activities, anxiety, insecurities, unpredictable living conditions, and perceived lack of control [44]. Our results demonstrate that SDoH improved during the pandemic, which may be attributed to VACHS providing enhanced healthcare delivery mechanisms for treating MHCs among PRVs. VACHS implemented initiatives focused on telehealth mental health care, PRVs virtual care, Veteran engagement efforts, healthcare education, and provision of electronic devices for accessing mental health care. However, SDoH had no impact on the clinical exacerbation of MHCs among SARS-CoV-2 infected PRVs or on increased suicidality. SDoH are independent risk factors that may have a differential impact on PTSD and SUD. Additional studies are needed to evaluate the implications of SDoH on MHCs by comparing PRVs and civilian communities in Puerto Rico.

## Conclusions

In summary, we have demonstrated an exacerbation of symptoms in individuals with both PTSD and SUD who were infected with SARS-CoV-2 among the PRVs during various catastrophic events, namely hurricanes Maria and Irma, the earthquakes, and the pandemic. A limitation of this study lies in the lower incidence of SARS-CoV-2 infections among PRVs with preexisting MHCs. To further solidify the current findings regarding SARS-CoV-2 infection as a risk factor for MHCs, a future large-scale multi-center study might be necessary. Additionally, larger sample-sized future studies are required to comprehensively understand the role of suicide risk factors, SDoH, and the impact of SARS-CoV-2 infection. These factors could contribute to the neuroinflammatory cascade that leads to the exacerbation of mental health issues and consequently HRS. In this study, we not only highlight how mental health can be influenced by the consequences of external factors stemming from the pandemic but also emphasize the potential direct impact of SARS-CoV-2 infection itself. This impact may have contributed to the clinical exacerbation of PTSD and SUD among the PRVs. The prevalence of SARS-CoV-2 infected patients with mental health conditions was of a fixed sample size due to their occurrence during the pandemic/study periods. These cases require mental health evaluation and treatment awareness and resource allocation for such studies is essential since we cannot discount the exacerbation of their MHCs. Therefore, the need for a striking balance between equitable access to mental health treatment resources becomes a critical challenge especially when the overall incidence is low but when the clinical impact is very substantially significant. This study also contributes to ethical considerations that are required for an equitable distribution of mental health service support for PRVs and intervention to mitigate the risk of SARS-CoV-2 infection and COVID-19 pandemic-induced mental health exacerbation.

## Data Availability

Original data that was generated from our research s provided within the manuscript after analysis was performed in the form of tables 1 to 4. The data provided is de-identified and the study was approved by the institutional review board of VACHS, San Juan, PR.
